# Supplementary material for: Nasal Spray Disinfectant for Respiratory Infections Based on Functionalized Silver Nanoparticles: A Physicochemical and Docking Approach
Source: Nanomaterials (Basel). 2025 Mar 31;15(7):533. doi: 10.3390/nano15070533 (PMC11990716; doi:10.3390/nano15070533)
Supplement: Supplementary file 1 [file nanomaterials-15-00533-s001.zip › nanomaterials-3540927-supplementary.pdf]

Nasal Spray Disinfectant for Respiratory Infections Based on Functionalized Silver Nanoparticles: A Physicochemical and Docking Approach

Benjamín Valdez-Salas[1], Jorge Salomón Salvador-Carlos[1,*]. Ernesto Alonso Valdez-Salas[2], Ernesto Alonso Beltrán-Partida[1], Jhonathan Castillo-Saenz[1], Mario Curiel-Álvarez[1], Daniel Gonzalez-Mendoza[3] and Nelson Cheng[4]

[1]Core Facilities of Chemistry and Advanced Materials, Instituto de Ingeniería, Universidad Autónoma de Baja California, Calle de La Normal S/N and Boulevard Benito Juárez, Mexicali 21100, México.
[2]Centro Médico Ixchel. Av. Nicolás Bravo 270, 21000. Mexicali, Baja California, México.
[3]Instituto de Ciencias Agrícolas, Universidad Autónoma de Baja California, Carretera a Delta s/n, Ejido Nuevo Leon, Mexicali 21705, Baja California, Mexico.
[4]Magna International Pte Ltd., 10 H Enterprise Road, Singapore 629834, Singapore.

[*]Corresponding author: jsalvador@uabc.edu.mx

Content

Table S1. Information of crystals used for molecular docking study. 3

Table S2. Wavenumbers (in cm-1) of selected vibrations of complex, AgNPs, and NSD, corresponding to the peaks marked in Figure 2d. 4

Table S3. ACE2-Hydrophobic interactions 5

Table S4. ACE2-Hydrogen bonds 6

Table S5. ACE2-Salt bridges 7

Table S6. Wild-Hydrophobic interactions 8

Table S7. Wild-Hydrogen bonds 9

Table S8. Wild-π-stacking 10

Table S9. Wild-π-cation interactions 11

Table S10. Wild-Salt bridges 12

Table S11. Delta-Hydrophobic interactions 13

Table S12. Delta-Hydrogen bonds 14

Table S13. Delta-Salt bridges 15

Table S14. Omicron-Hydrophobic interactions 16

Table S15. Omicron-Hydrogen bonds 17

Table S16. Omicron-π-stacking 18

Table S17. Omicron-Salt bridge 19

Table S1. Information of crystals used for molecular docking study.

Crystal	Description	Method	Resolution (Å)	Reference
6M0J	ACE2	X-ray diffraction	2.43	[1]
6M0J	Wild	X-ray diffraction	2.43	[1]
7V8B	Delta	Cryo-electron microscopy	3.20	[2]
7T9L	Omicron	Cryo-electron microscopy	2.66	[2]

Table S2. Wavenumbers (in cm-1) of selected vibrations of complex, AgNPs, and NSD, corresponding to the peaks marked in Figure 2d.

TA-SC	TA-SA-T80	AgNPs	NSD	Description
3364	3364	3364	3364	ν(O-H)
2924	2924	2924	2924	ν(C-H)
2854	2854	2854	2854	ν(C=O)
-	1736	1734	-	ν(C=O)
1576	1572	1586	1586	ν(C=O)
1392	1390	1398	1398	ν(C-OH)
1264	1262	1260	1260	ν(C-H)
-	1104	-	1114	ν(C-O-C)

For all interactions, we performed the study in Protein-Ligand Interaction Profiler website [3].

Table S3. ACE2-Hydrophobic interactions

Index	Residue	Amino acid	Distance	Ligand atom	Protein atom
1	26A	LYS	3.99	5947	74
2	33A	ASN	3.27	5981	149
3	89A	GLN	3.39	6041	710

 https://doi.org/10.3390/nano15070533

4	90A	ASN	3.91	6030	722
5	93A	VAL	3.91	6016	753
6	389A	PRO	3.46	6021	3672

Table S4. ACE2-Hydrogen bonds

Index	Residue	Amino acid	Distance H-A	Distance D-A	Donor angle	Protein donor	Side chain	Donor atom	Acceptor atom
1	27A	THR	1.96	2.50	115.02	No	Yes	5948 [O3]	86 [O3]
2	30A	ASP	2.95	3.75	144.46	Yes	Yes	118 [O3]	5949 [O3]
3	30A	ASP	3.55	3.88	101.11	Yes	No	110 [Nam]	5971 [O3]
4	33A	ASN	1.87	2.86	163.00	Yes	Yes	152 [Nam]	5972 [O3]
5	34A	HIS	3.18	3.52	100.61	Yes	Yes	166 [Npl]	5978 [O2]
6	90A	ASN	2.43	3.37	153.60	Yes	Yes	725 [Nam]	6008 [O2]
7	92A	THR	2.56	3.06	112.66	Yes	Yes	743 [O3]	6008 [O2]
8	96A	GLN	3.05	3.96	149.13	Yes	Yes	785 [Nam]	6017 [O2]
9	390A	PHE	2.16	2.96	133.67	Yes	No	3675 [Nam]	5982 [O2]
10	393A	ARG	3.10	3.90	135.83	Yes	Yes	3719 [Ng+]	6024 [O2]

Table S5. ACE2-Salt bridges

Index	Residue	Amino acid	Distance	Protein positive	Ligand group	Ligand atom
1	26A	LYS	3.56	Yes	Carboxylate	5937, 5939
2	26A	LYS	4.76	Yes	Carboxylate	5983, 5985
3	26A	LYS	3.99	Yes	Carboxylate	5933, 5935
4	26A	LYS	5.49	Yes	Carboxylate	5935, 6006
5	34A	HIS	5.05	Yes	Carboxylate	5967, 5972

Table S6. Wild-Hydrophobic interactions

Index	Residue	Amino acid	Distance	Ligand atom	Protein atom
1	449E	TYR	3.27	1938	1128
2	455E	LEU	2.90	1904	1205
3	489E	TYR	3.09	1967	1534
4	489E	TYR	3.60	1960	1536
5	493E	GLN	3.98	1944	1577
6	505E	TYR	3.98	1984	1689

Table S7. Wild-Hydrogen bonds

Index	Residue	Amino acid	Distance H-A	Distance D-A	Donor angle	Protein donor	Side chain	Donor atom	Acceptor atom
1	453E	TYR	2.34	3.04	129.68	Yes	Yes	1179 [O3]	1902 [O2]
2	489E	TYR	2.16	2.53	101.56	Yes	Yes	1539 [O3]	1920 [O3]
3	489E	TYR	2.12	2.53	104.62	No	Yes	1920 [O3]	1539 [O3]

4	493E	GLN	2.62	3.39	132.68	Yes	Yes	1582 [Nam]	1945 [O2]
5	493E	GLN	2.91	3.68	132.64	Yes	Yes	1585 [Nam]	1898 [O2]
6	494E	SER	1.96	2.94	159.04	Yes	No	1588 [Nam]	1945 [O2]
7	496E	GLY	2.89	3.86	159.48	Yes	No	1610 [Nam]	1934 [O3]
8	496E	GLY	2.88	3.75	154.98	No	No	1934 [O3]	1614 [O2]
9	501E	ASN	3.51	3.85	101.67	Yes	Yes	1663 [Nam]	1978 [O3]
10	502E	GLY	1.69	2.58	143.68	Yes	No	1666 [Nam]	1987 [O2]

Table S8. Wild-π-stacking

Index	Residue	Amino acid	Distance	Angle	Offset	Stacking type	Ligand atoms
1	505E	TYR	3.75	2.89	1.08	P	1983, 1984, 1985, 1986, 1988, 1990

Table S9. Wild-π-cation interactions

Index	Residue	Amino acid	Distance	Offset	Protein charged	Ligand group	Ligand atoms
1	417E	LYS	4.57	1.43	Yes	Aromatic	1903, 1904, 1905, 1906, 1908, 1910

Table S10. Wild-Salt bridges

Index	Residue	Amino acid	Distance	Protein positive	Ligand group	Ligand atom
1	403E	ARG	4.84	Yes	Carboxylate	1923, 1925
2	417E	LYS	5.45	Yes	Carboxylate	1907, 1912

Table S11. Delta-Hydrophobic interactions

Index	Residue	Amino acid	Distance	Ligand atom	Protein atom
1	456A	PHE	3.49	2083	1251
2	484A	GLU	3.70	2045	1527
3	489A	TYR	3.30	2072	1573
4	490A	PHE	3.40	2063	1590
5	490A	PHE	3.44	2046	1585

Table S12. Delta-Hydrogen bonds

Index	Residue	Amino acid	Distance H-A	Distance D-A	Donor angle	Protein donor	Side chain	Donor atom	Acceptor atom
1	417A	LYS	2.48	3.03	112.97	Yes	Yes	851 [N3+]	2084 [O2]
2	450A	ASN	2.46	3.26	142.59	No	Yes	1995 [O3]	1167 [O2]
3	452A	ARG	2.27	3.14	141.69	Yes	Yes	1196 [Ng+]	1982 [O3]
4	485A	GLY	1.73	2.56	140.55	No	No	2016 [O3]	1535 [O2]
5	490A	PHE	3.30	4.10	136.21	Yes	No	1580 [Nam]	1971 [O2]
6	493A	GLN	2.93	3.49	115.66	Yes	No	1617 [Nam]	1969 [O2]
7	494A	SER	2.33	3.29	158.12	Yes	No	1620 [Nam]	1975 [O2]

Table S13. Delta-Salt bridges

Index	Residue	Amino acid	Distance	Protein positive	Ligand group	Ligand atom
1	452A	ARG	4.64		Carboxylate	1980, 1985

Table S14. Omicron-Hydrophobic interactions

Index	Residue	Amino acid	Distance	Ligand atom	Protein atom
1	456A	PHE	3.52	2017	1256
2	475A	ALA	3.42	2028	1459
3	489A	TYR	3.93	2027	1578
4	493A	ARG	3.82	2069	1615
5	505A	HIS	3.79	2100	1739

Table S15. Omicron-Hydrogen bonds

Index	Residue	Amino acid	Distance H-A	Distance D-A	Donor angle	Protein donor	Side chain	Donor atom	Acceptor atom
1	408A	ARG	2.15	3.09	151.38	Yes	Yes	782 [Ng+]	2056 [O3]
2	408A	ARG	2.01	2.83	135.79	Yes	Yes	779 [Ng+]	2058 [O2]
3	453A	TYR	1.95	2.79	147.33	Yes	Yes	1217 [O3]	1988 [O2]
4	475A	ALA	3.11	3.78	127.63	No	No	2033 [O3]	1458 [O2]
5	494A	SER	2.05	3.01	155.05	Yes	No	1627 [Nam]	2070 [O2]
6	496A	SER	2.19	2.66	108.93	Yes	Yes	1655 [O3]	2071 [O3]
7	498A	ARG	2.71	3.17	107.45	Yes	Yes	1680 [Ng+]	2077 [O2]
8	498A	ARG	2.67	3.15	108.69	Yes	Yes	1683 [Ng+]	2077 [O2]
9	502A	GLY	2.01	2.97	155.74	Yes	No	1716 [Nam]	2101 [O2]

10	505A	HIS	1.94	2.89	153.59	Yes	Yes	1741 [Npl]	2088 [O2]

Table S16. Omicron-π-stacking

Index	Residue	Amino acid	Distance	Angle	Offset	Stacking type	Ligand atoms
1	489A	TYR	5.39	66.77	1.77	T	2016, 2017, 2018, 2019, 2021, 2023
2	501A	TYR	4.21	22.84	1.45	P	2093, 2094, 2095, 2096, 2098, 2100
3	505A	HIS	4.82	66.46	1.20	T	2093, 2094, 2095, 2096, 2098, 2100

Table S17. Omicron-Salt bridge

Index	Residue	Amino acid	Distance	Protein positive	Ligand group	Ligand atom
1	493A	ARG	4.49	Yes	Carboxylate	2059, 2061
2	505A	HIS	4.16	Yes	Carboxylate	2086, 2091

References

(1) Lan, J.; Ge, J.; Yu, J.; Shan, S.; Zhou, H.; Fan, S.; Zhang, Q.; Shi, X.; Wang, Q.; Zhang, L.; Wang, X. Structure of the SARS-CoV-2 Spike Receptor-Binding Domain Bound to the ACE2 Receptor. *Nature* **2020**, *581* (7807), 215–220. https://doi.org/10.1038/s41586-020-2180-5.
(2) Mannar, D.; Saville, J. W.; Zhu, X.; Srivastava, S. S.; Berezuk, A. M.; Tuttle, K. S.; Marquez, A. C.; Sekirov, I.; Subramaniam, S. SARS-CoV-2 Omicron Variant: Antibody Evasion and Cryo-EM Structure of Spike Protein–ACE2 Complex. *Science* **2022**, *375* (6582), 760–764. https://doi.org/10.1126/science.abn7760.
(3) Adasme, M. F.; Linnemann, K. L.; Bolz, S. N.; Kaiser, F.; Salentin, S.; Haupt, V. J.; Schroeder, M. PLIP 2021: Expanding the Scope of the Protein–Ligand Interaction Profiler to DNA and RNA. *Nucleic Acids Research* **2021**, *49* (W1), W530–W534. https://doi.org/10.1093/nar/gkab294.

Disclaimer/Publisher's Note: The statements, opinions and data contained in all publications are solely those of the individual author(s) and contributor(s) and not of MDPI and/or the editor(s). MDPI and/or the editor(s) disclaim responsibility for any injury to people or property resulting from any ideas, methods, instructions or products referred to in the content.
